# Making Use of Patient-Reported Outcome Measures for Haemorrhoidal Disease in Clinical Practice: A Perspective

**DOI:** 10.3389/fsurg.2021.728532

**Published:** 2021-08-25

**Authors:** Sara Z. Kuiper, Merel L. Kimman, Håvard D. Rørvik, Gunnar Olaison, Stephanie O. Breukink

**Affiliations:** ^1^Department of Surgery, School of Nutrition and Translational Research in Metabolism (NUTRIM), Maastricht University, Maastricht, Netherlands; ^2^Department of Clinical Epidemiology and Medical Technology Assessment, Care and Public Health Research Institute (CAPHRI), Maastricht University Medical Centre, Maastricht, Netherlands; ^3^Department of Acute and Digestive Surgery, Haukeland University Hospital, Bergen, Norway; ^4^Department of Surgery, Holbæk Hospital, Holbæk, Denmark; ^5^Department of Surgery, Maastricht University Medical Centre, Maastricht, Netherlands; ^6^Department of Surgery, School for Oncology and Developmental Biology (GROW), Maastricht University, Maastricht, Netherlands

**Keywords:** patient-reported outcome measures, core outcome set, haemorrhoidal disease, hemorrhoids, patient perspective

## Abstract

Haemorrhoidal disease (HD) affects millions of people around the world and for most it is a recurring problem. Increasingly, clinicians broaden their focus on the patient's experiences with haemorrhoidal symptoms, including their impact on daily life. The patient's experience can be assessed using a patient-reported outcome measure (PROM). A PROM facilitates a deeper understanding of the disease-burden and allows a clinician to obtain information directly from the patients about their experiences with the ailment. Over the last years, PROMs have shown their additional role to traditional outcomes for several diseases and have earned their place in the daily consultation room. In order to improve and personalize the treatment of HD, we endorse the use of validated PROMs in clinical care.

## Introduction

Haemorrhoidal disease (HD) is the most common proctological disease with prevalence rates of up to 44% within the general population (1). HD has troubled humankind since ancient times and considerably hampers a patient's quality of life (2). Patients report several restrictions or adjustments to be made in daily life: “Because of the massive blood loss, I could not function normally any more. I did not dare to go anywhere, not to a party, not to my son's soccer match” (3). Furthermore, HD may impair a patient's intimate relationship and sexuality: “(…) my sex life, I do think it is difficult, because of the flap coming out of my anus” (3–5).

In the past, traditional clinical outcomes such as “recurrence of disease” have been valued the most in clinical decision making and to denote treatment success. However, the emphasis is gradually shifting to the patients' perspective and patients' experience with symptoms of HD. This is also acknowledged in the recently developed European core outcome set (COS) for HD, by identifying patient-reported symptoms as the primary core outcome for clinical HD studies (6). A COS is a consensus-based agreed minimum set of outcomes that should be measured and reported in all clinical trials of a specific disease (7). A patient-reported outcome measure (PROM) captures a deeper understanding of the disease-burden by obtaining information directly from the patient about their experiences with the illness without interpretation by the healthcare professional or others (8). PROMs can focus on symptoms, functional outcomes, or broader concepts such as health-related quality of life. They have initially been utilized in health research and are now increasingly being used in daily clinical practice to support treatment decision making and follow-up care (9, 10).

This paper offers a perspective on the importance of PROM use in patients suffering from HD.

## The Rise of PROMs in Healthcare

Over the last years the additional value of using PROMs in clinical practice has been demonstrated and their popularity in various healthcare settings is rising (11). The systematic use of PROMs enhances communication and decision-making between doctor and patient, functioning as a ground layer in the process of shared decision-making (SDM) (5). SDM is a method where clinicians and patients decide together on the best treatment option through effective communication (12). In this process, evidence-based knowledge of the clinician and the individual patient's preferences, values and needs are taken into account. An important benefit of this approach is that it promotes value-based health care (VBHC). VBHC is defined as “the creation and operation of a health system that explicitly prioritizes health outcomes which matter to patients relative to the costs of achieving this outcome” (13). Hence, transforming the clinician's question of “What is the matter?” into “What matters to you?” Which is exactly what a PROM aims to capture.

A distinction can be made between generic and disease-specific PROMs. Generic PROMs are not bound to a specific disease and can measure the quality of life or health profile of any patient. Examples are the European Quality of Life—five dimensions (EQ-5D-5L) (14) and the Short Form 36 (SF-36) (15). Disease-specific PROMs evaluate the patient's outcomes related to a particular condition. In the field of gastroenterology alone, there are over 100 disease-specific PROMs available (16). Some successful examples are the PROM for peptic ulcers (PU-PROM) (17) and the Inflammatory Bowel Disease Questionnaire (IBDQ) (18).

## Clinical Decision Making in Haemorrhoidal Disease

Many therapeutic options have been developed for the treatment of HD. The first management step for HD concerns basic treatment, including laxatives, a high fiber diet and topical treatments. If basic treatment fails, patients are usually referred to the hospital for surgical consultation. Besides outpatient procedures like rubber band ligation and sclerotherapy, surgical options can also be considered, i.e., sutured or stapled haemorrhoidopexy, or traditional excisional surgery (19). The preferred procedure to treat HD mostly depends on the anal pathology of HD, categorized by the Goligher grade. The Goligher grading system categorizes HD into four grades: Grade I are hemorrhoids that do not prolapse; grade II are hemorrhoids that prolapse but reduce spontaneously; grade III are hemorrhoids that prolapse but have to be reduced manually; and grade IV are hemorrhoids that prolapse and cannot be reduced manually (20). Yet, the classification has several limitations. Firstly, a validation study of the Goligher classification has never been performed and thus it is unclear whether this classification is the most appropriate way to categorize HD and guide treatment strategies. Secondly, in the classification, only the symptom “prolapse” is included and is assessed by a clinician. Yet, patients with HD can suffer from other symptoms, i.e., blood loss, soiling, itching and pain (21). The Goligher classification does not consider these associated symptoms of HD (22). As a consequence, the broader impact of the disease on the patient may not be fully understood. While PROMs are ideally suited to assess this broader impact, they are not yet common practice in the treatment pathway for HD. There is indeed great potential in the usage of PROMs, not only to inform a treatment decision, but also to evaluate treatment success and the patient's satisfaction with the treatment (23). It is known that consensus on treatment success can differ substantially between healthcare professionals and patients, given that the doctor observes the disease, yet the patient experiences the symptoms (3, 24).

## Current PROMs for Haemorrhoidal Disease

Over time, several PROMs for HD have been developed. In the recent systematic review of Jin et al., a clear overview of available PROMs for HD is presented (25). Among the five PROMs discussed, the Haemorrhoid and Fissure Quality of Life Questionnaire (HEMO-FISS-QoL) extents its population to patients with fissures (4) and the Proctological Symptom Scale (PSS) aims to address the symptoms of patients with all sorts of proctological ailments (26). Not mentioned in the systematic review but nevertheless a valid and reliable tool to evaluate disease burden of the proctological patient, is the Proctoprom (5). Similar to the PSS, the Proctoprom is a PROM that takes the full range of proctology patients into account instead of focussing on HD. Expanding the population of the PROM can facilitate the swiftness of implementation but may reduce its relevance and validity. Hence, we recommend using a PROM which is specifically developed for use in a HD population.

Jin et al. discusses three of such PROMs for HD in his systematic review. The Sodergren score of Pucher et al. is specifically for HD patients and comprises of three items: intensity of pain, pruritus, and prolapse (27). The score is based on a scoring system developed by Nyström et al. that originally contained five symptoms: pain, pruritus, prolapse, bleeding, and soiling (28). The Sodergren score excluded the latter two symptoms based on a regression analysis and validation of the scoring system in a small sample of HD patients. For these two scores, no consensus-based standards for designing and reporting validation research were used (29). The Haemorrhoid Severity Score (HSS) of Lee et al. uses the same symptomatology as Nyström and has assessed the psychometric aspect “responsiveness” in two large multi-center, randomized controlled trials (RCT) (30–32). The fifth PROM described in the Jin review is the Haemorrhoidal Disease Symptom Score and Short Health Scale for Haemorrhoidal Disease (HDSS and SHS-HD) developed by Rørvik et al. (33). Validation of the HDSS and SHS-HD was built on consensus-based standards for designing and reporting validation research. This score encompasses all five symptoms as introduced by Nyström barring a modification of the question on prolapse. The scoring system by Nyström assesses how frequently the patient needs to reduce the prolapse, restricting the question to patients with a Goligher grade III. In contrast, the HDSS asks how often the patient experiences a swelling or prolapse in the anus, making the question applicable to Goligher grades II-IV. A short health scale was added to probe the impact of the HD symptoms on daily life, as well as impact on mental and general well-being. A quality of life instrument complements the use of a HD-symptom score since it provides a more generic view on how the symptoms are perceived in a day-to-day setting. The HDSS SHS-HD by Rørvik et al. has shown satisfactory results when methodologically assessed and can be used in the consultation room.

Finally, a PROM for HD has recently been introduced as an important outcome measure for two large clinical trials in The Netherlands (34, 35). The PROM-Haemorrhoidal Impact and Satisfaction Score (PROM-HISS) is the first PROM for HD developed in dialogue with patients suffering from HD (3). It was developed in response to the COS and measures the same HD symptoms as the previously mentioned scoring systems of both Nyström and Rørvik: prolapse, blood loss, pain, soiling and itching. Furthermore, it includes a quality of life question probing the impact of the HD symptoms on performing daily activities. A final question evaluates the patient's satisfaction with treatment related to reducing their symptom burden. A fundamental validation study of the PROM-HISS is currently being performed.

## Future Directions in Haemorrhoidal Disease PROMs

Symptoms of a disease may be interpreted differently by the patient who experiences them than the clinician who observes them. Especially in a proctological disease like HD, where patients may feel shame or embarrassment, safeguarding an open conversation is crucial. In clinical practice, a HD PROM can support this discussion and indicate the issue or symptom which is most important for the patient. A PROM facilitates the process of SDM and functions as a valuable tool to encourage a patient-centered approach. Consequently, the patient will feel heard and understood, resulting in effective conversations, and providing a more detailed insight into the patients' experiences with HD. Discussion points are not limited to medical subjects, but can also cover the impact of symptoms on daily activities. It is of paramount importance that the patient feels that the conversation is about him and his needs. Exploring the disease burden and treatment expectations of patients with help of a PROM improves patient satisfaction with care (36). Additionally, in our experience as clinicians, the conversation with the patient is facilitated when a PROM has been completed before the consultation since it can quickly identify issues of concern to the patient (37).

We strongly advice the use of a PROM in clinical HD practice, in particular a PROM that has been developed following recommended guidelines and has been validated. Suggestions are the HDSS, and once established valid, the PROM-HISS. These symptom-focused PROMs are ideally complemented with a HD quality of life tool such as the SHS-HD.

Starting to use a PROM in the consultation room will maybe take some time getting used to but in the long run it will increase the quality of patient care. Because a patient who receives a personalized treatment, is a more satisfied patient.

## Conclusion

The patient's perspective is vital for clinical decision making. Systematic assessment of patient-reported outcomes using PROMs provides a thorough understanding of the symptom burden and experienced health of patients and can inform a tailored clinical HD treatment. We recommend the use of the HDSS and, once validated, the PROM-HISS, preferably combined with an HD quality of life tool suchlike the SHS-HD.

## Data Availability Statement

The original contributions presented in the study are included in the article/supplementary material, further inquiries can be directed to the corresponding author/s.

## Author Contributions

SK wrote the manuscript. MK, HR, GO, and SB provided guidance and a critical review. All authors jointly conceptualized the article and discussed the aspects of the topic to be addressed.

## Conflict of Interest

The authors declare that the research was conducted in the absence of any commercial or financial relationships that could be construed as a potential conflict of interest.

## Publisher's Note

All claims expressed in this article are solely those of the authors and do not necessarily represent those of their affiliated organizations, or those of the publisher, the editors and the reviewers. Any product that may be evaluated in this article, or claim that may be made by its manufacturer, is not guaranteed or endorsed by the publisher.

## References

[1] Riss S Weiser FA Schwameis K Riss T Mittlbock M Steiner G. The prevalence of hemorrhoids in adults. Int J Colorectal Dis. (2012) 27:215–20. 10.1007/s00384-011-1316-3 21932016

[2] Sun Z Migaly J. Review of hemorrhoid disease: presentation and management. Clin Colon Rectal Surg. (2016) 29:22–9. 10.1055/s-0035-1568144 26929748 PMC4755769

[3] Tol RRV Kimman ML Breukink SO Kuiper SZ Melenhorst J Stassen LP. Experiences of patients with haemorrhoidal disease-a qualitative study. J Coloproctol. (2019) 39:41–7. 10.1016/j.jcol.2018.10.005

[4] Abramowitz L Bouchard D Siproudhis L Trompette M Pillant H Bord C. Psychometric properties of a questionnaire (HEMO-FISS-QoL) to evaluate the burden associated with haemorrhoidal disease and anal fissures. Colorectal Dis. (2019) 21:48–58. 10.1111/codi.14393 30171745 PMC7379620

[5] Vander Mijnsbrugge GJ Molenaar C Buyl R Westert G van der Wees PJ. How is your proctology patient really doing? Outcome measurement in proctology: development, design and validation study of the Proctoprom. Tech Coloproctol. (2020) 24:291–300. 10.1007/s10151-020-02156-2 32112248

[6] van Tol RR Kimman ML Melenhorst J Stassen LPS Dirksen CD Breukink SO. European society of coloproctology core outcome set for haemorrhoidal disease: an international Delphi study among healthcare professionals. Colorectal Dis. (2019) 21:570–80. 10.1111/codi.14553 30628177

[7] Gorst SL Prinsen CAC Salcher-Konrad M Matvienko-Sikar K Williamson PR Terwee CB. Methods used in the selection of instruments for outcomes included in core outcome sets have improved since the publication of the COSMIN/COMET guideline. J Clin Epidemiol. (2020) 125:64–75. 10.1016/j.jclinepi.2020.05.021 32470621

[8] Black N. Patient reported outcome measures could help transform healthcare. BMJ. (2013) 346:f167. 10.1136/bmj.f167 23358487

[9] Damman OC Jani A de Jong BA Becker A Metz MJ de Bruijne MC. The use of PROMs and shared decision-making in medical encounters with patients: An opportunity to deliver value-based health care to patients. J Eval Clin Pract. (2020) 26:524–40. 10.1111/jep.13321 31840346 PMC7155090

[10] Field J Holmes MM Newell D. PROMs data: can it be used to make decisions for individual patients? A narrative review. Patient Relat Outcome Meas. (2019) 10:233–41. 10.2147/PROM.S156291 31534379 PMC6681163

[11] Meadows KA. Patient-reported outcome measures: an overview. Br J Community Nurs. (2011) 16:146–51. 10.12968/bjcn.2011.16.3.146 21378658

[12] Elwyn G Frosch D Thomson R Joseph-Williams N Lloyd A Kinnersley P. Shared decision making: a model for clinical practice. J Gen Intern Med. (2012) 27:1361–7. 10.1007/s11606-012-2077-6 22618581 PMC3445676

[13] Vetter TR Uhler LM Bozic KJ. Value-based healthcare: preoperative assessment and global optimization (PASS-GO): improving value in total joint replacement care. Clin Orthop Relat Res. (2017) 475:1958–62. 10.1007/s11999-017-5400-z 28600689 PMC5498398

[14] Brooks R. EuroQol: the current state of play. Health Policy. (1996) 37:53–72. 10.1016/0168-8510(96)00822-6 10158943

[15] Brazier JE Harper R Jones NM O'Cathain A Thomas KJ Usherwood T. Validating the SF-36 health survey questionnaire: new outcome measure for primary care. BMJ. (1992) 305:160–4. 10.1136/bmj.305.6846.160 1285753 PMC1883187

[16] Khanna P Agarwal N Khanna D Hays RD Chang L Bolus R. Development of an online library of patient-reported outcome measures in gastroenterology: the GI-PRO database. Am J Gastroenterol. (2014) 109:234–48. 10.1038/ajg.2013.401 24343547 PMC4275098

[17] Liu N Lv J Liu J Zhang Y. The PU-PROM: A patient-reported outcome measure for peptic ulcer disease. Health Expect. (2017) 20:1350–66. 10.1111/hex.12575 28636163 PMC5689228

[18] Guyatt G Mitchell A Irvine EJ Singer J Williams N Goodacre R. A new measure of health status for clinical trials in inflammatory bowel disease. Gastroenterology. (1989) 96:804–10. 10.1016/0016-5085(89)90905-0 2644154

[19] van Tol RR Kleijnen J Watson AJM Jongen J Altomare DF Qvist N. European Society of ColoProctology: guideline for haemorrhoidal disease. Colorectal Dis. (2020) 22:650–62. 10.1111/codi.14975 32067353

[20] Goligher JC Duthie HL Nixon HH. Surgery of the Anus, Rectum, and Colon. 3rd ed. London: Baillière Tindall (1975).

[21] van Tol RR Melenhorst J Dirksen CD Stassen LPS Breukink SO. Protocol for the development of a Core Outcome Set (COS) for hemorrhoidal disease: an international Delphi study. Int J Colorectal Dis. (2017) 32:1091–4. 10.1007/s00384-017-2833-5 28501943 PMC5486628

[22] Gallo G Martellucci J Sturiale A Clerico G Milito G Marino F. Consensus statement of the Italian society of colorectal surgery (SICCR): management and treatment of hemorrhoidal disease. Tech Coloproctol. (2020) 24:145–64. 10.1007/s10151-020-02149-1 31993837 PMC7005095

[23] Damman OC Verbiest MEA Vonk SI Berendse HW Bloem BR de Bruijne MC. Using PROMs during routine medical consultations: the perspectives of people with Parkinson's disease and their health professionals. Health Expect. (2019) 22:939–51. 10.1111/hex.12899 31199574 PMC6803413

[24] Montgomery AA Fahey T. How do patients' treatment preferences compare with those of clinicians? Qual Health Care. (2001) 10 Suppl 1:i39–43. 10.1136/qhc.0100039 11533437 PMC1765739

[25] Jin J Xia W Connolly A Hill AG. Symptom based scoring for haemorrhoidal disease: a systematic review. Colorectal Dis. (2020) 22:1518–27. 10.1111/codi.15253 32639663

[26] Kraemer M Kara D Rzepisko M Sayfan J. A simple tool to evaluate common disorders: validation of a “proctological symptom scale”. Int J Colorectal Dis. (2015) 30:679–82. 10.1007/s00384-015-2160-7 25694137 PMC4544484

[27] Pucher PH Qurashi M Howell AM Faiz O Ziprin P Darzi A. Development and validation of a symptom-based severity score for haemorrhoidal disease: the Sodergren score. Colorectal Dis. (2015) 17:612–8. 10.1111/codi.12903 25603811

[28] Nystrom PO Qvist N Raahave D Lindsey I Mortensen N. Randomized clinical trial of symptom control after stapled anopexy or diathermy excision for haemorrhoid prolapse. Br J Surg. (2010) 97:167–76. 10.1002/bjs.6804 20035531

[29] Terwee CB Prinsen CAC Chiarotto A Westerman MJ Patrick DL Alonso J. COSMIN methodology for evaluating the content validity of patient-reported outcome measures: a Delphi study. Qual Life Res. (2018) 27:1159–70. 10.1007/s11136-018-1829-0 29550964 PMC5891557

[30] Lee MJ Morgan J Watson AJM Jones GL Brown SR. A validated severity score for haemorrhoids as an essential prerequisite for future haemorrhoid trials. Tech Coloproctol. (2019) 23:33–41. 10.1007/s10151-019-01936-9 30725242 PMC6394714

[31] Brown SR Tiernan JP Watson AJM Biggs K Shephard N Wailoo AJ. Haemorrhoidal artery ligation versus rubber band ligation for the management of symptomatic second-degree and third-degree haemorrhoids (HubBLe): a multicentre, open-label, randomised controlled trial. Lancet. (2016) 388:356–64. 10.1016/S0140-6736(16)30584-0 27236344 PMC4956910

[32] Watson AJ Hudson J Wood J Kilonzo M Brown SR McDonald A. Comparison of stapled haemorrhoidopexy with traditional excisional surgery for haemorrhoidal disease (eTHoS): a pragmatic, multicentre, randomised controlled trial. Lancet. (2016) 388:2375–85. 10.1016/S0140-6736(16)31803-7 27726951 PMC5269572

[33] Rorvik HD Styr K Ilum L McKinstry GL Dragesund T Campos AH. Hemorrhoidal disease symptom score and short health ScaleHD: new tools to evaluate symptoms and health-related quality of life in hemorrhoidal disease. Dis Colon Rectum. (2019) 62:333–42. 10.1097/DCR.0000000000001234 30451751

[34] Kuiper SZ Dirksen CD Kimman ML Van Kuijk SMJ Van Tol RR Muris JWM. Effectiveness and cost-effectiveness of rubber band ligation versus sutured mucopexy versus haemorrhoidectomy in patients with recurrent haemorrhoidal disease (Napoleon trial): Study protocol for a multicentre randomized controlled trial. Contemp Clin Trials. (2020) 99:106177. 10.1016/j.cct.2020.106177 33080380

[35] Dekker L Han-Geurts IJM van Dieren S Bemelman WA. HollAND trial: comparison of rubber band ligation and haemorrhoidectomy in patients with symptomatic haemorrhoids grade III: study protocol for a multicentre, randomised controlled trial and cost-utility analysis. BMJ Open. (2021) 11:e046836. 10.1136/bmjopen-2020-046836

[36] Nelson EC Eftimovska E Lind C Hager A Wasson JH Lindblad S. Patient reported outcome measures in practice. BMJ. (2015) 350:g7818. 10.1136/bmj.g7818 25670183

[37] Graupner C Breukink SO Mul S Claessens D Slok AHM Kimman ML. Patient-reported outcome measures in oncology: a qualitative study of the healthcare professional's perspective. Support Care Cancer. (2021) 29:5253–61. 10.1007/s00520-021-06052-9 33655412 PMC8295145

